# Supplementary material for: Toll-like Receptors and Cytokine Modulation by Goat Milk Extracellular Vesicles in a Model of Intestinal Inflammation
Source: Int J Mol Sci. 2023 Jul 4;24(13):11096. doi: 10.3390/ijms241311096 (PMC10341995; doi:10.3390/ijms241311096)
Supplement: Supplementary file 1 [file ijms-24-11096-s001.zip › ijms-2438024-supplementary.pdf]

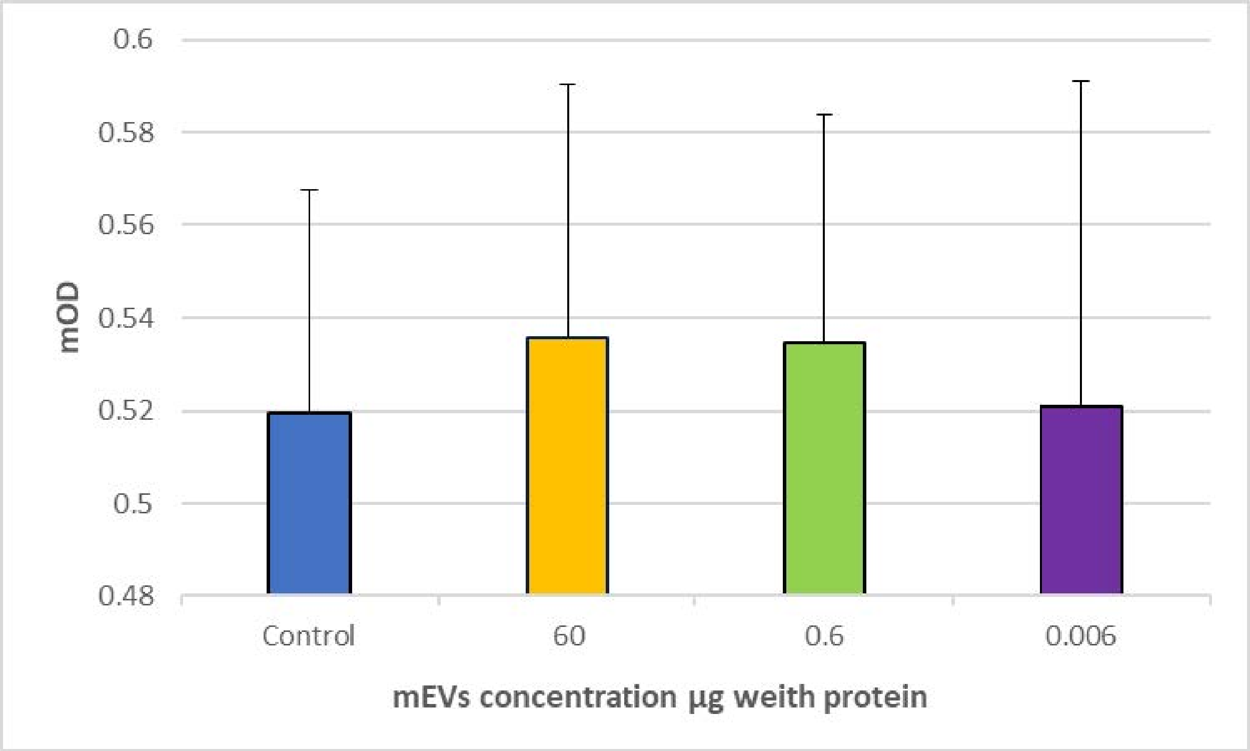

Figure S1. Viability of IPEC-J2 after mEVs exposure at 48h. The different concentrations of mEVs did not determine a significant difference in terms of cell viability after 48 h of treatment. Data are expressed as optical density (OD) ± SD. Differences were evaluated through the Kruskal-Wallis test and applying the post-doc Dunn's Multiple Comparison Test. Statistical significance: $p < 0.05$.

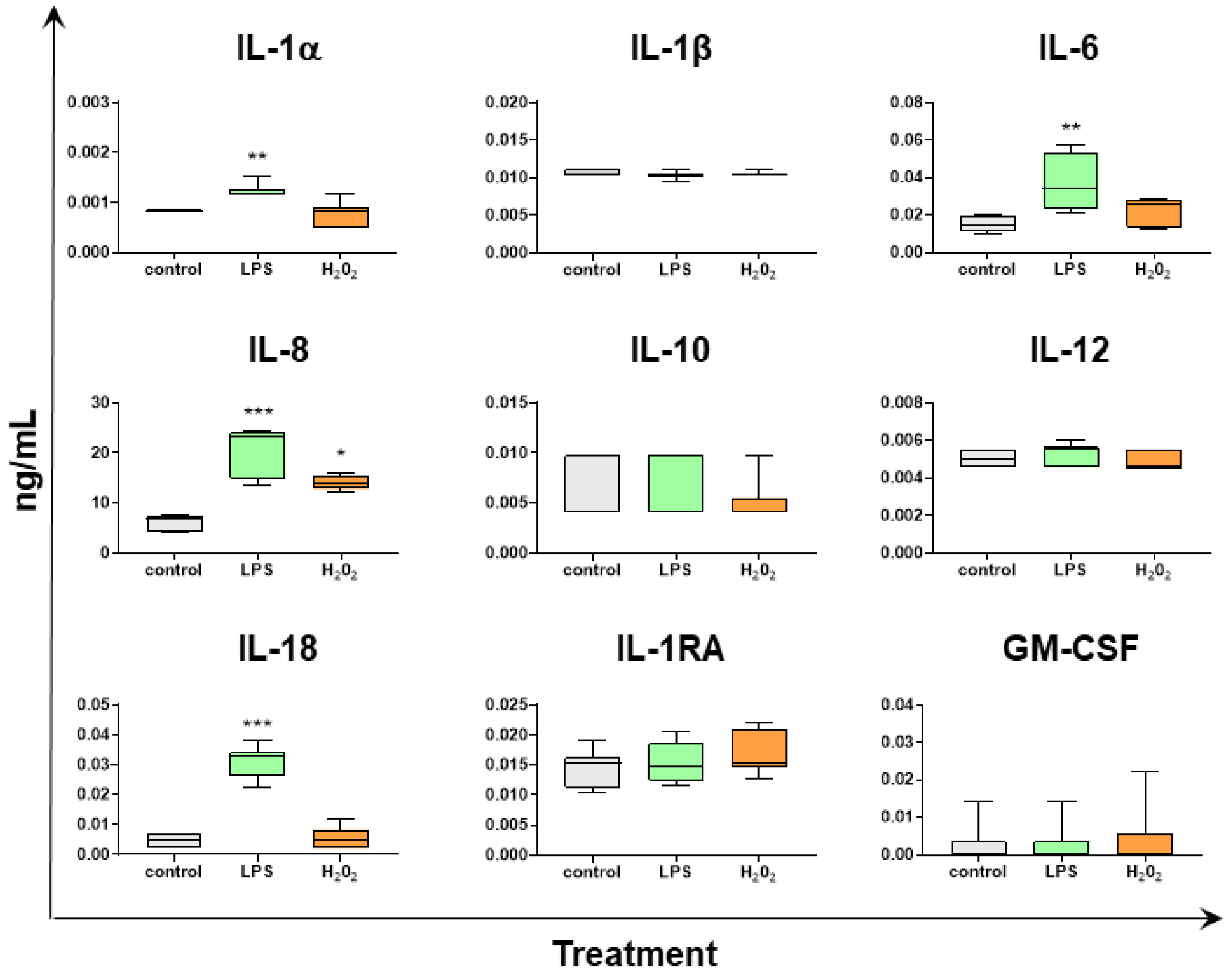

Figure S2. Cytokine release by IPEC-J2 after 24 h of treatment with LPS or H_2O_2. IPEC-J2 were left untreated (control, grey), stimulated with LPS (green) or H_2O_2 (orange). After, culture supernatants were collected, and levels of several cytokines were determined through multiplex ELISA. Data are presented as box-and -whisker plots displaying median and interquartile range (boxes) and minimum and maximum values (whiskers). Differences (control vs LPS; control vs H_2O_2) were evaluated through ANOVA followed by Dunnett's multiple comparison test or a Kruskal-Wallis test followed by Dunn's multiple comparison test; * $p<0.05$, ** $p<0.01$ and *** $p<0.001$.

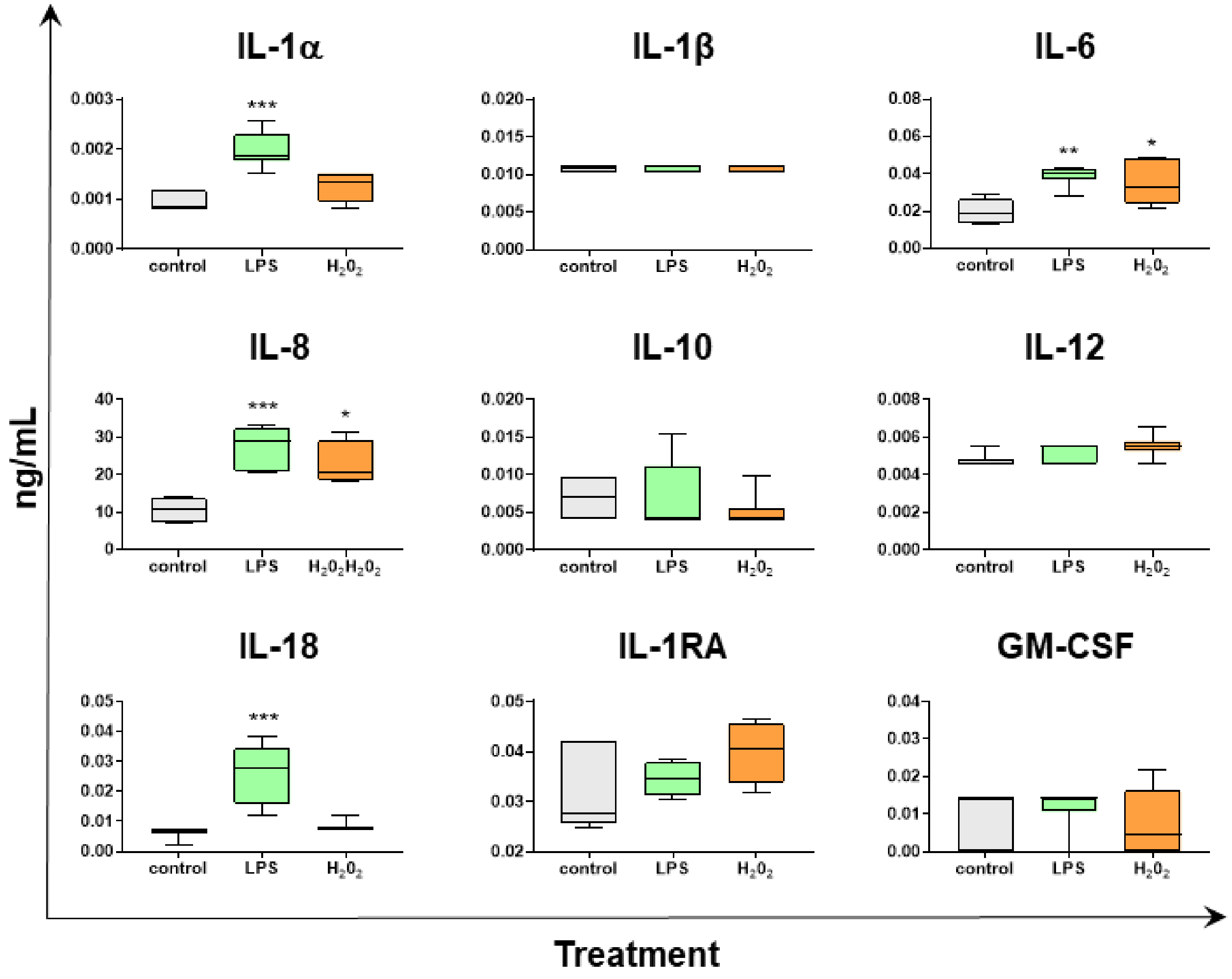

Figure S3. Cytokine release by IPEC-J2 after 48 h treatment with LPS or H_2O_2. IPEC-J2 were left untreated (control, grey), stimulated with LPS (green) or H_2O_2 (orange). After 48 h, culture supernatants were collected, and levels of several cytokines were determined through multiplex ELISA. Data are presented as box-and -whisker plots displaying median and interquartile range (boxes) and minimum and maximum values (whiskers). Differences (control vs LPS; control vs H_2O_2) were evaluated through ANOVA followed by Dunnett's multiple comparison test or a Kruskal-Wallis test followed by Dunn's multiple comparison test; * $p<0.05$, ** $p<0.01$ and *** $p<0.001$.

Table S1. Primer set sequences for target and reference genes.

Gene	Primer sequences	Amplicon length	Source
IL12A	For - 5′ - ATGCCTCAACCACTCCCAAA – 3′<br>Rev – 5′ – TGTGCTGGTTTTATCTTTGGTGA – 3′	135	[12]
IL12B	For - 5′ - TCAGGGACATCATCAAACCA – 3′<br>Rev – 5′ – GAACACCAAACATCAGGGAAA – 3′	140	[58]
EBI3	For - 5′- CAACGTCACAGCCATCCAC – 3′<br>Rev – 5′ – GGTTTCCACTGCACCCAA – 3′	140	[12]
IL6	For - 5′ - TGGCTACTGCCTTCCCTACC – 3′<br>Rev – 5′ – CAGAGATTTTGCCGAGGATG – 3′	131	[59]
CXCL8	For - 5′- TTCGATGCCAGTGCATAAATA – 3′<br>Rev – 5′ – CTGTACAACCTTCTGCACCCA – 3′	175	[59]
IL18	For - 5′ - CGTGTTTGAGGATATGCCTGATT - 3<br>Rev – 5′ - TGGTTACTGCCAGACCTCTAGTGA - 3′	106	[59]
TNFA	For - 5′ - TGCCTACTGCACTTCGAGGTTATC – 3′<br>Rev – 5′ – GTGGGCGACGGGCTTATCTG – 3′	125	[60]
TGFB1	For - 5′ - CGC GTG CTA ATG GTG GAA AG – 3′<br>Rev - 5′ - CCG ACG TGT TGA ACA GCA TA – 3′	87	[12]
NOS2	For - 5′ - CGTTATGCCACCAACAATGG – 3′<br>Rev – 5′ – AGACCCGGAAGTCGTGCTT – 3′	84	[61]
MUC2	For - 5′ - GCAAAGACCTCTAAGATGGCC – 3′<br>Rev – 5′ – AGACCACACTCAGCCTCAG – 3′	132	[12]
MMP9	For - 5′ - TGT GGA CCA GAT GTT CCC C – 3′<br>Rev – 5′ – AGT CCA CCT GAT TCA CCT CG – 3′	130	[12]
TLR1	For - 5′ - AGA TTT CGT GCC ACC CTA TG – 3′<br>Rev – 5′ – CCT GGG GGA TAA ACA ATG TG – 3′	276	[61]
TLR2	For - 5′ - CGG CTT CCA AGG ATG GAG AAA - 3′<br>Rev – 5′ - TCC AGA GAG TTG ACC TTG CAG - 3′	71	[61]
TLR3	For - 5′ - TGAAGAACTTGATTTCCTTGGCA – 3′<br>Rev – 5′ – GGCATGAAAACACCCTGGAG – 3′	93	[61]
TLR4	For - 5′ - TGGCAGTTTCTGAGGAGTCATG – 3′<br>Rev – 5′ –CCGCAGCAGGGACTTCTC – 3′	71	[59]
TLR5	For - 5′ - TCAAAGATCCTGACCATCACA – 3′<br>Rev. – 5′ – CCAGCTGTATCAGGGAGCTT – 3′	59	[59]
TLR7	For - 5′-GTGGAAATTGCCCTCGTTGT – 3′<br>Rev – 5′ –GATGGATCTGTAGGGGAGCA – 3′	77	[61]
TLR8	For - 5′ - AAGACAACCAGTTACGTGAAATACC – 3′<br>Rev –5′ – GGGTGTTAAAAGATAATGACAGCAC – 3′	236	[62]
TLR9	For - 5′ - AGGACTTCATGCCAAACTGC – 3′<br>Rev – 5′ – CGAGCAAACATCTCCGACTG – 3′	90	[61]
DEFB1	For - 5′ - TGCCACAGGTGCCGATCT - 3′<br>Rev – 5′ - CTGTTAGCTGCTTAAGGAATAAAGGC - 3′	80	[59]
DEFB4A	For - 5′ - CCAGAGGTCCGACCACTA – 3′<br>Rev – 5′ – GGTCCCTTCAATCCTGTT – 3′	87	[59]
MYD88	For - 5′ - GCA GCT GGA ACA GAC CAA CT - 3′<br>Rev - 5′ - GTG CCA GGC AGG ACA TCT - 3′	62	[63]
NFKB1	For - 5′ - CCCATGTAGACAGCACCACCTATGAT - 3′<br>Rev - 5′ - ACAGAGGCTCAAAGTTCTCCACCA - 3′	131	[63]
RELA	For - 5′ –CGAGAGGAGCACGGATACCA - 3<br>Rev - 5′ –GCCCCGTGTAGCCATTGA - 3′	61	[63]
IFNA1	For - 5′ - GGCTCTGGTGCATGAGATGC - 3′<br>Rev - 5′ - GCCTTCTTCCTGAATCTGTCTCA - 3′	546	[64]

	IFNB	For - 5′ - AGTTGCCTGGGACTCCTCAA - 3′<br>Rev - 5′ - CCTCAGGGACCTCGAAGTTCAT – 3′	59	[65]
	IRF3	For - 5′ - GGGAAGGAGGCGTGTTCGAC - 3′<br>Rev - 5′ - ACCAGAGGGTGTAGCGTGGT - 3′	92	[63]
Ref.	*GAPDH*	For - 5′ - ATGGTGAAGGTCGGAGTGAA – 3′<br>Rev – 5′ – AGTGGAGGTCAATGAAGGGG – 3′	61	[12]
